# The Paradoxical Effects of COVID-19 Event Strength on Employee Turnover Intention

**DOI:** 10.3390/ijerph19148434

**Published:** 2022-07-10

**Authors:** Hui Deng, Wenbing Wu, Yihua Zhang, Xiaoyan Zhang, Jing Ni

**Affiliations:** 1 School of Economics and Management, Beijing Jiaotong University, Beijing 100044, China; 19113068@bjtu.edu.cn (H.D.); wbwu@bjtu.edu.cn (W.W.); 2 Graduate School of Education and Psychology, Pepperdine University, Los Angeles, CA 90045, USA; yihua.zhang@pepperdine.edu; 3 School of Business, Qingdao University, Qingdao 266071, China; neevj_7@163.com

**Keywords:** COVID-19 event strength, perceived external employability, perceived organizational growth, turnover intention, organizational identification

## Abstract

As a global pandemic, the novel coronavirus (COVID-19) has brought enormous challenges to employees and organizations. Although numerous existing studies have highlighted that the COVID-19 pandemic is a stressful event and empirically proved its detrimental effect on employee turnover intention, few scholars have noted that this pandemic can deteriorate the external economic and employment environment simultaneously, which may further complicate employees’ intentions to leave or stay in the current organization. Drawing on event system theory and social cognitive theory, this study aims to uncover two potential cognitive mechanisms of the complex impact of COVID-19 event strength on employee turnover intention. To examine the proposed model, this study employed a three-wave and time-lagged research design and collected data from a sample of 432 employees of four Chinese companies from different industries. The findings indicated that COVID-19 event strength was negatively related to perceived external employability, and ultimately curbed employee turnover intention. Yet, COVID-19 event strength also negatively predicted perceived organizational growth, thus influencing employees to exhibit intentions to quit. Moreover, organizational identification not only attenuated the positive effect of perceived external employability on turnover intention but also amplified the negative impact of perceived organizational growth on turnover intention. Further, organizational identification moderated the indirect effects of COVID-19 event strength on turnover intention through perceived external employability and perceived organizational growth. This study provided a comprehensive insight into scholars’ understanding of the COVID-19 downstream outcomes.

## 1. Introduction

As a global epidemic, the outbreak of the novel coronavirus (COVID-19), seen as “a humanitarian crisis” and “the greatest test” since the 1940s [1], has caused a great slowdown in global economic activities. According to recent estimates by the International Labor Organization [2], the level of working hours is expected to be 4.2% below the pre-pandemic level, equivalent to 123 million full-time jobs. This indicates that the COVID-19 pandemic not only posed significant challenges to organizations and influenced their long-term development [3], but also potentially interfered with employees’ conduct in the workplace. For instance, numerous studies have identified the detrimental effects of the COVID-19 pandemic on several employee workplace behaviors, including organizational deviance [4], avoidance coping behavior [5], and workplace cheating behavior [6]. Notably, some scholars illuminated that, during such a hard era, how to retain qualified employees also represents a major challenge for organizational practice [7,8]. Recent studies have empirically investigated that as a stressful event, the COVID-19 pandemic has damaged the relationships between employees and organizations and stimulated employees’ negative psychological states [9], which in turn may increase their turnover intention [10]. However, these studies still have several limitations.

First, previous studies have proved that employees’ intentions to leave have been increased due to the COVID-19 crisis [10,11]. Indeed, taking into account the external labor market, the deteriorating economic environment has not only reduced job opportunities but also forced a large number of employees to lose their existing jobs [12]. Thus, this stressful event can also lead to intense competition in the job market, which may further facilitate employees to place a high value on their current job. As such, it is conceivable that the decision-making process for employees to leave or stay in an organization during the epidemic is complicated. Nevertheless, extant studies have rarely addressed the potential inhibitory effect of COVID-19 on employee turnover intention or uncovered this complex process. Second, from the perspective of work stress, a majority of existing studies have primarily revealed employees’ psychological mediators to explain how COVID-19 leads to surges in turnover intention among employees, including emotional exhaustion [10], job insecurity [13], and psychological anxiety [14]. However, the mediating mechanisms that explain COVID-19 detrimental impacts have not been fully explored. Remarkably, little scholarly attention has been paid to exploring whether such an event would exacerbate employees’ turnover intentions by triggering their negative social cognitions. Hence, to fill these gaps, it is essential to develop a more comprehensive model to explore whether, how, and when COVID-19 event strength affects employee turnover intention.

In this study, we thus employ event system theory [15] and social cognitive theory [16] as our overarching frameworks and propose a paradoxical cognitive mechanism in accounting for the complex relationship between COVID-19 and employee turnover intention. Specifically, this study theorizes that the overall strength of the COVID event may threaten employees’ cognitive appraisals of their career development and reduce subsequent intentions to leave current organization. Thus, as an important indicator of employees’ potential for career development [17], perceived external employability, which refers to employees’ beliefs about their competence in finding new employment in external labor market [18], is imported to explain the mechanism linking COVID-19 to employees’ turnover intention in this study. Moreover, the undesirable impacts of the COVID-19 crisis may not only involve employees’ career development but also spread to the organizations’ overall development [19]. During the COVID-19 outbreak, the deteriorating external environment would hinder organizational sustainability development [20], which may elicit employees’ negative cognitive evaluations of the growth of current organization and engender their intentions to leave subsequently. This study thus speculates that perceived organizational growth may be another pivotal cognitive mechanism in accounting for how COVID-19 event strength affects employee turnover intention.

In addition, social cognitive theory states that affected by situational factors, employees with the same cognitive level behave differently [21]. Organizational identification refers to employees’ perceptions of oneness with their organizations [22]. Strong identified employees are inclined to be psychologically attached to their current organizations [23], which may alter the strength of perceived external employability and perceived organizational growth impact employee turnover intention. Therefore, this study proposes an integrated framework to explain the complex impacts of COVID-19 event strength on employee turnover intention with perceived external employability and organizational growth as mediators and organizational identification as a moderator.

In this article, we first elaborate on how COVID-19 event strength is complicatedly associated with employee turnover intention through perceived external employability and perceived organizational growth. Then, we detail how organizational identification moderates the two paths described above. After that, we report the findings of an empirical study conducted on four companies in China and finally conclude by discussing the theoretical and practical implications.

## 2. Theoretical Background and Research Hypotheses

### 2.1. Event System Theory, COVID-19 Event Strength

Event system theory [15] states that the time, space, and strength of events that happen in life and work have dynamic impacts on individuals’ psychological and behavioral responses. In this study, we focus on exploring the aftereffects of COVID-19 event strength, reflecting the degree to which individuals perceive this pandemic as meaningful and impactful [4,15]. Based on event system theory, COVID-19 epidemic strength includes three attributes: novelty, disruption, and criticality [15]. Among these attributes, COVID-19 event novelty refers to the extent to which employees consider that the epidemic varies from previous ones in that it creates new changes in job demands and makes it difficult for established procedures to direct employees’ behaviors at work. COVID-19 event disruption reflects the extent to which employees believe that the epidemic changes routine activities of organizations or themselves. COVID-19 event criticality refers to employees’ perceptions of priority of organizations or themselves in responding to the epidemic and the impacts of COVID-19 on their goals.

To date, several recent studies have applied event system theory to explore the downstream outcomes of COVID-19 event strength in the workplace [4,24,25,26]. Due to the suddenness of COVID-19 and its widespread impacts, this pandemic has obviously generated enormous challenges for employees’ work and life. As such, this study suggests that event system theory can be an appropriate framework to unveil the mechanism of COVID-19 event strength on employee turnover intention.

### 2.2. COVID-19 Event Strength, Perceived External Employability, and Turnover Intention

Perceived external employability is described as an employee’s self-perception about finding a new job with another employer [17]. Previous research has revealed that potential for career advancement and flexibility, expertise compared with others, and obtained development activities can be powerful signals for employees to assess their employability [17,27]. In the context of COVID-19, the labor market slack caused by this ongoing epidemic remains significant [10], which is also leading to the emergence of a formidable and challenging environment for employee job transition [5]. Therefore, this study posits that COVID-19 event strength can be a crucial indicator of predicting employee perceived external employability.

Social cognitive theory suggests that there is a triangular interaction among the social environment, individual cognition, and their behaviors [16]. Individuals tend to form social cognitions to guide their subsequent behaviors through interpreting the information obtained from social environment [16,28]. Given that the confluence of novelty, disruption, and criticality reflects the overall strength of the COVID-19 event [15], this study further speculates that employees who consider the COVID-19 pandemic as a vital event are prone to shape negative perceptions of external employability in unique ways by its three characteristics.

First, the COVID-19 event novelty focuses on provoking unexpected changes to work processes and procedures, further placing new job requirements for employees [15]. Thus, when employees perceive COVID-19 as novel, they may find it difficult to meet the evolving job demands with their usual work skills and experiences [4]. Therefore, such a sense of incompetence may signal to employees that their career advancements are severe and render their negative perceptions of external employability. Second, COVID-19 event disruption stresses the subversion of organizational and employees’ routine activities due to the response and risks (e.g., lockdown, quarantine, traffic restrictions, and infection) associated with the outbreak [15,29]. Strict containment measures have hampered economic growth, especially increasing downside risks in the labor market [5,30]. Thus, employees experiencing high levels of COVID-19 event disruption are more likely to interpret acquiring employment opportunities in the labor market as tough, resulting in low levels of perceived external employability. Third, the COVID-19 event criticality reflects the priority of employee response to this event and its impact on employees’ attainment of their long-term career success [5,15]. Employees who perceive the COVID-19 pandemic as critical will to pay more energy and time coping with this crisis. Constantly changing quarantine measures also have drastically reduced employees’ job training, resulting in their original long-term career planning falling apart [4]. Accordingly, they are more likely to have increased concerns about their career stability and development, and then perceive scarce external employability [17]. Combining the above arguments about the novelty, disruption, and criticality of the COVID-19 event, this study thus proposes:

**Hypothesis** **1a.** *COVID-19 event strength is negatively associated with perceived external employability.*

Based on social cognitive theory [16], perceived low external employability may be a key indicator in curbing employees’ intention to quit. In particular, employees with low levels of external employability are liable to lack confidence in their capability to seize alternative career development opportunities in the external labor market [31]. Such negative perceptions of job alternatives may elicit employees to attach more importance to their current job and be reluctant to leave their organizations [17,32]. In addition, the postulated positive relationship between perceived external employability and turnover intention has been explicitly confirmed by several empirical studies [27,33], which also provides evidence for supporting the above argument. Consequently, this study proposes the following:

**Hypothesis** **1b.** *Perceived external employability is positively associated with turnover intention.*

Based on the above discussion, this study further proposes that the COVID-19 event strength may decrease employees’ perceived external employability, which in turn reduces their intentions to quit. This study thus assumes:

**Hypothesis** **1c.** *Perceived external employability mediates the relationship between COVID-19 event strength and turnover intention.*

### 2.3. COVID-19 Event Strength, Perceived Organizational Growth, and Turnover Intention

As an important indicator of overall organizational sustainability performance, organizational growth refers to the development of physical attributes as well as human resources within an organization [34,35]. Considerable research on organizational growth has revealed its positive impacts on the reputation of an organization, employees’ productivity, and behaviors [35,36,37]. Given that the rapid spread of COVID-19 has brought uncertainties to the economic environment and interrupted organizations’ business sustainability [7], this study thus argues that COVID-19 event strength may be a crucial predictor for employees to estimate the growth of current organization.

In line with event system theory and social cognitive theory, COVID-19 event strength may decrease employee perceived organizational growth in three paths. First, as outlined above, employees who perceive COVID-19 as novel are prone to interpret that their organizations lack crisis management abilities and experiences in coping with this epidemic [38,39]. As a result, they are inclined to raise concerns about business continuity and sustainable performance of their organizations, which evokes negative thoughts about organizational growth [4,40]. Second, with regard to COVID-19 event disruption, scholars have found that in response to the outbreak, many organizations have had to suspend their daily production operations and commercial activities, which may damage their sustainable development [4,7]. In this case, employees who experience high levels of COVID-19 event disruption are more likely to develop a negative assessment of the current organizational growth [37]. Third, when the COVID-19 epidemic is highly critical, organizations need to prioritize resources for the epidemic prevention and control in their daily operations [25]. While most attention is mobilized to respond to the epidemic, resources allocated to organizational business development will be limited [41], impeding the attainments of their long-term goals. As such, employees tend to render more concerns about the growth of their current organizations during this epidemic. Taken together, this study postulates the following:

**Hypothesis** **2a.** *COVID-19 event strength is negatively associated with perceived organizational growth.*

In accordance with social cognitive theory [16], this study further speculates the increase in employees’ turnover intention as a key reaction to perceived low levels of organizational growth. Specifically, as Feldman [42] noted, employees manage their careers by focusing on long-term organizational growth. Employees who perceive low levels of organizational growth are more likely to deem that their current organizations have difficulties in providing them with adequate job resources and opportunities for future career growth [43]. As a result, they may develop intentions to leave the current organization [44]. Moreover, when employees negatively estimate the outlook of their organizations, they are more likely to find it difficult to get a competitive salary in current organizations [45,46], which may further induce intentions to quit [47]. Based on the discussion above, this study then hypothesizes:

**Hypothesis** **2b.** *Perceived organizational growth is negatively associated with turnover intention.*

Combined with the above arguments, this study further predicts that the COVID-19 event strength may engender employees’ negative perceptions of the organizational growth, and subsequently provokes their turnover intentions. Hence, this study posits:

**Hypothesis** **2c.** *Perceived organizational growth mediated the relationship between COVID-19 event strength and turnover intention.*

### 2.4. The Moderating Role of Organizational Identification

Organizational identification reflects the extent to which organizational members perceive overlap between themselves and organizational membership [22]. Numerous previous studies have stated that employees who possess high levels of organizational identification are committed to adjusting their behaviors in accordance with the organization’s expectations, such as increasing organizational citizenship behaviors [48], job crafting [49], and creativity [50].

As social cognitive theory states [21,28], situational factors play determinant roles in affecting the strength of internalizing cognitive to behavior. Therefore, this study suggests that organizational identification can be regarded as a potential buffer for employees’ perceived external employability to influence turnover intention. Specifically, Karanika-Murray et al. [51] indicated that as an affective bond, organizational identification reflects the strength of the connection between employees and organizations. Compared to employees with low organization identification, strong identifiers have stronger affective attachments to the current organization. Such attachment would amplify employees’ psychological dependence on the organization [52,53], which is most notable when they have difficulty obtaining external employment opportunities. In this case, employees are less likely to generate intentions to quit. Moreover, Boon et al. [54] argued that employees with a strong sense of organizational identification tend to define themselves regarding their membership in the organization, and those employees are more likely to stay in their organization, especially when they are less able to obtain external employment opportunities. As such, this study argues that organizational identification may attenuate the detrimental effects of perceived external employability on turnover intention. Thus, the following hypothesis is postulated:

**Hypothesis** **3a.** *Organizational identification moderates the positive relationship between perceived external employability and turnover intention, such that the relationship is weakened more for employees with higher levels of organizational identification than employees with lower levels of organizational identification.*

Moreover, Ashforth et al. [55] suggested that employees who possess high levels of organizational identification are prone to value the whole organization’s interests more than their own interests. Thus, when organizationally identified employees perceived that severe external events (e.g., the COVID-19 crisis) would have negative impacts on the sustainability of organizational growth, they may be inclined to stay and take actions to improve the development of the current organization rather than quit. Previous research has also shown that organizational identification can be regarded as a pivotal factor affecting employees’ responses to stressful situations [56]. In this study, when facing a stressful circumstance in which the future of current organization is tough, employees with strong organizational identification are prone to develop confidence in the organization’s ability to get out of such a dilemma. As such, they are less likely to generate intentions to quit. Combined with the above arguments, it is logical to argue that employees who strongly identify themselves as belonging to the current organization are unwilling to leave, even if they have a pessimistic outlook on the organizational growth. Hence, this study hypothesizes the following:

**Hypothesis** **3b.** *Organizational identification moderates the negative relationship between perceived organizational growth and turnover intention, such that the relationship is strengthened more for employees with higher levels of organizational identification than employees with lower levels of organizational identification.*

### 2.5. An Integrative Model

To integrate the above hypotheses, this study proposes a moderated mediation framework in which organizational identification moderates the mediating effects of perceived external employability as well as perceived organizational growth on the relationship between COVID-19 event strength and turnover intention. Accordingly, this study proposes the following hypotheses:

**Hypothesis** **4a.** *Organizational identification moderates the mediating effect of perceived external employability on the relationship between COVID-19 event strength and turnover intention, such that the mediating effect is strengthened more for employees with lower levels of organizational identification than employees with higher levels of organizational identification.*

**Hypothesis** **4b.** *Organizational identification moderates the mediating effect of perceived organizational growth on the relationship between COVID-19 event strength and turnover intention, such that the mediating effect is strengthened more for employees with higher levels of organizational identification than employees with lower levels of organizational identification.*

**Figure 1:**
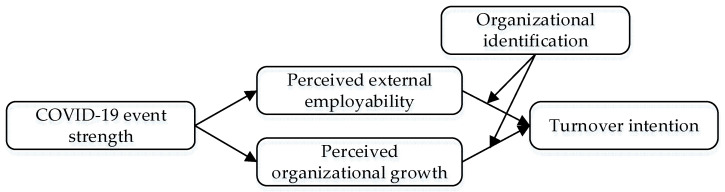
Research theoretical model.

Our theoretical model is displayed in Figure 1.

## 3. Methods and Measures

### 3.1. Sample and Procedure

Given that our study focused on exploring the impact of COVID-19 on employee turnover intention, we conducted a survey of four companies in different industries that were susceptible to the pandemic (e.g., catering and tourism). The data collection began in March 2022 and ended in April 2022. To comply with the targeted companies’ antiepidemic policy, we used online survey (Wenjuanxing) and meeting (VooV Meeting) platforms to conduct the survey. With the assistance of each company’s human resource department, we obtained an initial list of 544 participants. At the beginning of formal survey, this study introduced the purpose, anonymity, and confidentiality of our survey to all participants through the online meeting. Then, to ensure the authenticity and quality of data, we asked all participants to fill out the questionnaire by scanning the QR code with their WeChat and matched each of them with a unique five-digit password.

To alleviate concerns about common method bias, we collected data from three time points, maintaining a two-week interval for each wave. At Time 1, 492 participants reported COVID-19 event strength, organizational identification, perceived insider status, and provided basic demographic information. At Time 2, 454 participants rated their perceptions of external employability and organizational growth. At Time 3, 432 participants reported their intentions to leave the current organization. Finally, we received 432 valid samples, with an overall response rate of 79.41%. Table 1 illustrates the demographic statistics of final samples in detail.

### 3.2. Measures

The translation and back-translation approach suggested by Brislin [57] was applied to translate all measures from English to Chinese. Each item was measured on a seven-point Likert scale (1 = strongly disagree to 7 = strongly agree), unless otherwise specified.

*COVID-19 event strength.* COVID-19 event strength was measured using an eleven-item scale that included three dimensions: event novelty, event disruption, and event criticality [15,58,59]. A sample item is “This COVID-19 pandemic requires me to change the way I do my work”. To further explore whether these items can be aggregated into a composite score, this study conducted a second-order confirmatory factor analysis (CFA). The results indicated that the second-order CFA model can fit the data well (χ^2^ (41) = 1.046, RMSEA = 0.010, SRMR = 0.016, CFI = 0.999, TLI = 0.999). Thus, following the suggestions of Wong et al. [60] and Morgeson F. P. et al. [15], this study combined these items in an additive fashion to reflect the overall strength of the COVID-19 event.

*Perceived external employability.* Perceived external employability was assessed using a four-item scale from De Cuyper et al. [31]. A sample item is “It would not be very difficult for me to get an equivalent job in a different organization”.

*Perceived organizational growth.* This study adapted a three-item scale based on Haque et al.’s [35] and Lee and Ha-Brookshire’s [37] work to measure perceived organizational growth. A sample item is “During the COVID-19 pandemic, my current company has competitive advantages in its sales growth”.

*Turnover intention.* This study measured turnover intention with a four-item scale developed by Kelloway et al. [61]. A sample item is “I don’t plan to be in the current company much longer”.

*Organizational identification.* This study measured organizational identification using a six-item scale from Edwards and Peccei [62]. A sample item is “I feel strong ties with the current company”.

*Control Variables.* In alignment with prior research, this study included employees’ gender, age, educational level, years of work experience, type of current enterprise, position, and marital status, as control variables [5,10,24,37]. Moreover, this study controlled for employees’ perceived insider status, as it can affect their intention to remain in the current organization [63]. This study measured perceived insider status with a six-item scale developed by Stamper and Masterson [64]. A sample item is “I feel I am an ‘insider’ in the current organization”.

## 4. Results

### 4.1. Preliminary Analyses

As the data in this study were collected from a single source, we conducted the Harman single factor analysis to examine whether common method variance would affect the results. The results indicated that all the extracted factors accounted for 74.45% of the variance, with the first factor accounting for 26.71% of it, which was less than the cutoff value of 40%. Thus, this study is free from common method variance. Moreover, in line with Scott et al.’s work [65], we calculated ICC (1) values to examine whether there is sufficient variance across the four companies to warrant aggregation. The results indicated that the ICC (1) values were: for COVID-19 event strength = −0.002; for perceived external employability = −0.007; for perceived organizational growth = 0.00008; for turnover intention = 0.010; and for organizational identification = −0.001, which were all less than its threshold value of 0.12 [66]. Thus, it is conceivable that organization-related nesting effects were not significant within our data.

### 4.2. Confirmatory Factor Analysis

This study further performed confirmatory factor analysis with Mplus 8.3 to confirm the discriminant validity among the constructs. The results indicated that the model fitness indices of hypothesized model provided adequate fit to the data, with χ^2^ (512) = 1.229, RMSEA = 0.014, SRMR = 0.026, CFI = 0.996, TLI = 0.996. The standardized factor loading of each item, each construct’s average variance extracted (AVE), and composite reliability (CR) are presented in Table 2. The results showed that the values of factor loadings (ranged from 0.675 to 0.930), AVE (ranged from 0.564 to 0.842), and CR (ranged from 0.888 to 0.970) met the cut-off values of 0.50, 0.50, and 0.70, respectively [67,68]. Hence, it can be inferred that this study fulfilled the item reliability, convergence validity, and internal consistency requirements. The diagonal of Table 3 represents the square root of each variable’s AVE value, which is higher than its correlation with other variables, thereby suggesting qualified discriminant validity.

### 4.3. Descriptive Statistics

The descriptive statistics and inter-correlations among all study variables are depicted in Table 3. In general, the patterns of correlations were as expected. COVID-19 event strength was negatively related to perceived external employability (r = −0.326, *p* < 0.01) and perceived organizational growth (r = −0.300, *p* < 0.01). Further, perceived external employability was positively related to turnover intention (r = 0.161, *p* < 0.01), while perceived organizational growth was negatively related to turnover intention (r = −0.362, *p* < 0.01).

### 4.4. Hypotheses Testing

Mplus 8.3 was utilized to test all the proposed hypotheses. After introducing employee gender, age, educational level, years of work experience, type of current enterprise, position, marital status, and perceived insider status as control variables, we used hierarchical multiple regression to test the main, mediation, and moderation effects. In particular, we included organizational identification as an additional control variable for turnover intention before testing for moderation effects. As shown in Table 4, the results showed that COVID-19 event strength was significantly negatively related to perceived external employability (β = −0.331, *p* < 0.001, in Model 1), which in turn was positively associated with turnover intention (β = 0.105, *p* < 0.01, in Model 5). Thus, H1a and H1b were supported. Similarly, COVID-19 event strength had a significant and negative association with perceived organizational growth (β = −0.298, *p* < 0.001, in Model 2), and perceived organizational growth had a significant and negative association with turnover intention (β = −0.318, *p* < 0.001, in Model 6), thereby indicating support for H2a and H2b. Then, we adopt a bootstrap method to examine the indirect effect of COVID-19 event strength on turnover intention via perceived external employability and perceived organizational growth. Results in Table 5 indicate that the indirect effect of COVID-19 event strength on turnover intention via perceived external employability was significant (β = −0.050, 95% CI = [−0.090, −0.011]), supporting H1c. Similarly, H2c, which predicted that perceived organizational growth mediates the link between COVID-19 event strength and turnover intention, was also supported (β = 0.155, 95% CI = [0.095, 0.215]).

**Figure 2:**
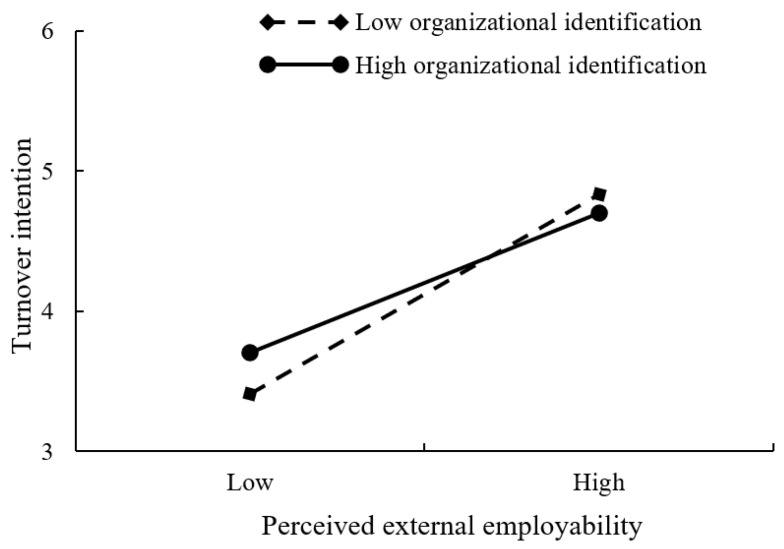
Interaction plot of organizational identification and perceived external employability on turnover intention.

**Figure 3:**
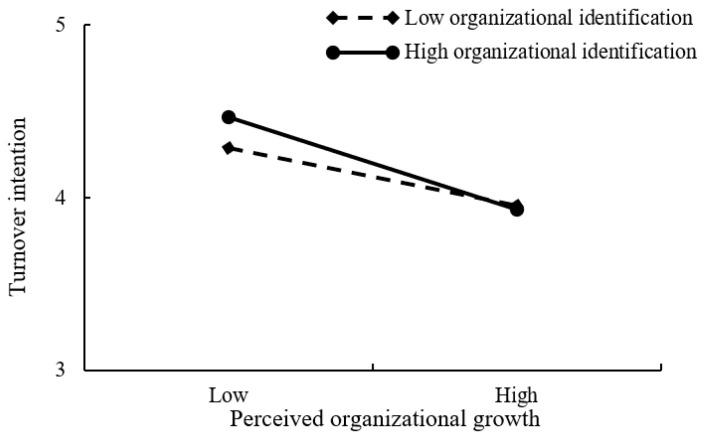
Interaction plot of organizational identification and perceived organizational growth on turnover intention.

Regarding the moderation effect of organizational identification, the results presented in Table 4 showed that the interaction of perceived external employability and organizational identification was negatively and significantly related to turnover intention (β = −0.072, *p* < 0.01, in Model 8). Following the suggestion of Aiken and West [69], this study further performed a simple slope analysis (see Figure 2) to depict the moderating role of organizational identification on the relationship between perceived external employability and turnover intention. The positive effect of perceived external employability on turnover intention was stronger when employees possessed lower levels of organizational identification. Likewise, the interaction of perceived organizational growth and organizational identification was negatively and significantly associated with turnover intention (β = −0.034, *p* < 0.05, in Model 8). As Figure 3 illustrates, the negative relationship between perceived organizational growth and turnover intention was stronger for employees with higher levels of organizational identification. Consequently, H3a and H3b were supported.

To further test the moderated mediating hypotheses, we performed a bootstrapping-based analytic approach. As shown in Table 6, for employees with high levels of organizational identification, the indirect effect of COVID-19 event strength on their turnover intention via perceived external employability was significant (indirect effect = −0.146, 95% CI = [−0.217, −0.086], excluding zero). For employees who possessed low levels of organizational identification, the indirect effect was stronger and significant (indirect effect = −0.209, 95% CI = [−0.319, −0.117], excluding zero). The difference between the two indirect effects was also significant (∆ indirect effect = 0.063, 95% CI = [0.021, 0.098], excluding zero). Thus, H4a was supported. Results in Table 6 also indicated that the indirect effect of COVID-19 event strength on turnover intention through perceived organizational growth was significantly positive only when organizational identification was high (indirect effect = 0.096, 95% CI = [0.007,0.136], excluding zero). Although when organizational identification was low, such an indirect effect was not significant (indirect effect = 0.059, 95% CI = [−0.024,0.129], including zero), the difference between the two indirect effects was significant (∆ indirect effect = 0.063, 95% CI = [0.021, 0.098], excluding zero), providing support for H4b.

## 5. Discussion

Drawing upon event system theory and social cognitive theory, this study proposed and examined how employees’ assessments of COVID-19 event strength affect their turnover intention in a complex manner with two paradoxical paths: a retention path through decreasing perceived external employability and a turnover path through reducing perceived organizational growth. As predicted, the findings revealed that COVID-19 event strength can decrease employees’ turnover intention by triggering their negative perceptions of external employability, but increasing their turnover intention through stimulating their perceived little organizational growth. Furthermore, the findings demonstrated the conditional indirect effect of COVID-19 event strength on turnover intention via perceived external employability and perceived organizational growth at separate values of organizational identification.

### 5.1. Theoretical Implications

This study contributes to the growing research on the COVID-19 event in several ways. First, our findings contribute to the existing research on the COVID-19 event by revealing its complex impacts on employee turnover intention. Previous research has consistently advocated that turnover intention was relatively prevalent during the COVID-19 pandemic [10,11,70]. In accord with prior scholars, this study illustrated a turnover path by introducing employee-perceived organizational growth as a mediator. Furthermore, this study also demonstrated the existence of another retention path. Specifically, this study found that the COVID-19 event strength can decrease employees’ perceptions of external employability, subsequently contributing to reducing the risk of turnover. Our work also responds to the call for exploring the challenges and opportunities COVID-19 poses to employee career management from a more holistic view [7].

Second, this research sheds light on perceived organizational growth as a pivotal mediating mechanism in accounting for how COVID-19 event strength provokes employee intention to leave. Most of the extant studies have explored the detrimental impacts of COVID-19 on employee behavior by conceptualizing it as employees’ work or psychological stressors [4,24,71]. However, little research has realized that the COVID-19 crisis can also be regarded as a stressful event that hinders organizational growth. This study advocates that the COVID-19 crisis has not only caused concerns for employees, but also posed numerous challenges to organizations. As such, employees would negatively evaluate the current organizational growth, and then are more likely to leave the organization. By uncovering the mediating role of perceived organizational growth, this study extends previous scholarly research on the adverse impacts of the COVID-19 pandemic [72].

Third, by considering organizational identification as a moderator, this study contributes to the research on boundary conditions of how the COVID-19 crisis affects employee intention to leave. Although previous studies have noticed the key factors that can affect the impacts of COVID-19, most of them examined the individual differences in personality such as resilience [73], mindfulness [9], and adaptive ability [74]. Following the call by Liu D. et al. [24], this research focused on a moderator regarding organization (i.e., organizational identification) and examined it as a buffer against the adverse effects of COVID-19 on employee turnover intention. Our findings demonstrate that employees with high levels of organizational identification are reluctant to voluntarily leave the current organization when they perceive low employability in the external labor market or are not optimistic about the sustainable growth of the current organization due to the COVID-19 event.

### 5.2. Practical Implications

This study not only examined the complex influences of COVID-19 event strength on employee turnover intention, but also provided viable solutions to management practice. First, this study found that COVID-19 event strength can lead to employees’ negative appraisals of the current organizational growth, and subsequent elicit intentions to leave. This finding reinforced the importance of fostering employees’ enthusiasm for continuous organization growth. For example, managers should put more effort into effective crisis management and enhancing organizational resilience and readiness for changes to cope with unanticipated events [35,75], which are conducive to achieving sustainable performance and growth of the organization [76].

Second, this study revealed that organizational identification can be an effective buffer against employees’ intention to quit the organization, most notable when they perceive strong external employability or negatively evaluate organizational sustainability performance. In line with previous research [56,77], our findings provide support for the importance of nurturing employees’ identification with the organization. Accordingly, to prevent employees’ strong intentions from leaving the organization, organizations should endeavor to provide opportunities for employees to identify with the current organization, including valuing long-term working relationships, maintaining fairness in organizational decision-making, and arranging psychological capital training programs [78].

### 5.3. Limitations and Future Research

This study also has several limitations that should be considered in future research. First, all constructs were inevitably measured from employees’ self-reporting, which may raise concerns about common method bias and thus overstate the relationship between variables [79]. Although this study collected data at three time points to mitigate this concern, we cannot make conclusions about causality from such a design. For instance, it is plausible that employees who negatively perceive their external employability may describe the COVID-19 pandemic as a strong event since it harms their employability. As such, longitudinal and multi-source designs are warranted in future work.

Second, the data collection in this study was primarily conducted from four companies in the limited industries that were vulnerable to the COVID-19 event, including catering, tourism, textile, and appliance industries. However, there are also a few industries where companies and employees have gained tremendous growth opportunities from COVID-19, such as healthcare and online offices. Indeed, more diversified research should be implemented to explore the variability of findings across industries further.

Third, this research only introduced organizational identification as a boundary condition of the influence of COVID-19 event strength on employee turnover intention and could not rule out other factors that may also contribute to modifying the relationship. Future research could include various factors as moderators in an effort to comprehensively account for the strength of the influence of the COVID-19 event on employees’ cognition. Furthermore, the decision-making process for employees to leave or stay in an organization during the COVID-19 epidemic is complicated. Future works are encouraged to examine other alternative underlying mechanisms that link COVID-19 event strength and employee turnover intention.

## 6. Conclusions

Drawing on event system theory and social cognitive theory, this study revealed the complex effects of COVID-19 event strength on employee turnover intention by introducing perceived external employability and perceived organizational growth as two potential mechanisms. Moreover, this study confirmed that the extent to which perceived external employability triggers employee turnover intention as well as the extent to which perceived organizational growth decreases employee turnover intention were both moderated by organizational identification. These findings add important value to the existing literature and provide a new insight for scholars, organizations, and employees to understand the effect of the COVID-19 pandemic.

## Figures and Tables

**Table 1:** Demographic statistics of the final sample (N = 432).

Variables	N	%	Variables	N	%
**Gender**			**Educational background**		
Male	236	54.63	High school degree or below	58	13.43
Female	196	45.37			
**Age**			Junior college degree	61	14.12
20–30	90	20.83	Bachelor degree	260	60.19
31–40	313	72.45	Master degree or above	53	12.26
>40	29	6.72	**Industry**		
**Years of work experience**			Catering	76	17.59
1–5	115	26.62	Tourism	127	29.40
6–10	243	56.25	Textile	137	31.71
>10	74	17.13	Appliance industries	92	21.30
**Position**			**Marital status**		
Employee	234	54.17	Unmarried	115	26.62
Supervisor	140	32.41	Married	269	62.27
Manager	58	13.42	Divorced	48	11.11

**Table 2:** Confirmatory factor analysis: Items and factor loadings (N = 432).

Variables	Items	Standardized Loadings	AVE	CR
COVID-19 event strength (CES)	CES1	0.764	0.564	0.934
	CES2	0.739		
	CES3	0.741		
	CES4	0.759		
	CES5	0.751		
	CES6	0.774		
	CES7	0.768		
	CES8	0.753		
	CES9	0.762		
	CES10	0.768		
	CES11	0.675		
Perceived external employability (PEE)	PEE1	0.820	0.664	0.888
	PEE2	0.823		
	PEE3	0.779		
	PEE4	0.837		
Perceived organizational growth (POS)	POG1	0.907	0.819	0.931
	POG2	0.896		
	POG3	0.912		
Turnover intention (TI)	TI1	0.877	0.765	0.929
	TI2	0.887		
	TI3	0.874		
	TI4	0.861		
Organizational identification (OI)	OI1	0.930	0.842	0.970
	OI2	0.900		
	OI3	0.909		
	OI4	0.925		
	OI5	0.916		
	OI6	0.925		
Perceived insider status (PIS)	PIS1	0.837	0.693	0.931
	PIS2	0.820		
	PIS3	0.826		
	PIS4	0.817		
	PIS5	0.852		
	PIS6	0.841		

**Table 3:** Means, standard deviations, and correlations of variables (N = 432).

Variables	Mean	SD	1	2	3	4	5	6	7	8	9	10	11	12	13
1. Gender	0.55	0.50													
2. Age	33.97	4.40	−0.006												
3. Education	2.71	0.85	0.125 **	0.032											
4. Experience	7.40	3.28	−0.068	0.696 **	−0.340 **										
5. Industry	2.67	1.10	0.032	0.102 *	−0.017	0.067									
6. Position	1.59	0.72	−0.006	0.236 **	0.025	0.181 **	0.207 **								
7. Marital status	1.84	0.56	−0.003	0.500 **	0.072	0.275 **	0.110 *	0.140 **							
8. PIS	3.65	1.24	0.031	0.013	−0.028	0.058	0.064	0.020	0.101 *	0.832					
9. CES	3.64	0.98	−0.062	0.015	0.010	−0.006	−0.009	−0.035	−0.041	−0.002	0.751				
10. PEE	4.12	1.32	−0.050	0.071	0.053	0.055	−0.003	0.075	0.017	−0.098 *	−0.326 **	0.815			
11. POG	3.69	1.74	0.009	−0.033	−0.010	0.014	0.032	−0.009	0.033	0.049	−0.300 **	0.026	0.905		
12. TI	4.16	1.50	−0.097 *	0.082	−0.032	0.036	−0.001	0.001	−0.020	−0.335 **	0.009	0.161 **	−0.362 **	0.875	
13. OI	4.02	1.85	0.124 *	−0.041	−0.004	−0.038	0.009	0.017	0.011	0.270 **	0.030	−0.077	0.066	−0.542 **	0.918

Note: * *p* < 0.05, ** *p* < 0.01 (two-tail test). The diagonal represents the discriminant validity.

**Table 4:** Regression results of main, mediation, and moderation effects (N = 432).

Variables	PEE	POG	TI					
	Model 1	Model 2	Model 3	Model 4	Model 5	Model 6	Model 7	Model 8
Gender	−0.073	−0.010	−0.028	−0.027	−0.023	−0.027	−0.079	−0.129
Age	0.049	−0.105	0.144 *	0.142 *	0.142 *	0.102	0.035	0.027
Education	0.079	0.023	−0.066	−0.066	−0.074	−0.059	−0.116	−0.107
Experience	0.048	0.080	−0.085	−0.084	−0.091	−0.054	−0.028	−0.025
Industry	−0.011	0.033	0.011	0.011	0.013	0.022	0.033	0.014
Position	0.049	−0.024	0.000	0.000	−0.007	−0.005	−0.028	−0.010
Marital status	−0.036	0.045	−0.040	−0.038	−0.038	−0.019	−0.050	−0.003
PIS	−0.094	0.040	−0.200 ***	−0.200 ***	−0.191 ***	−0.193 ***	−0.223 ***	−0.225 ***
CES	−0.331 ***	−0.298 ***		0.018			−0.076	−0.052
PEE					0.105 **		0.114 **	0.404 ***
POG						−0.318 ***	−0.290 ***	−0.145 *
OI			−0.482 ***	−0.482 ***	−0.477 ***	−0.463 ***	−0.370 ***	0.026
PEE × OI								−0.072 **
POG × OI								−0.034 *
R^2^	0.134 ***	0.098 ***	0.342 ***	0.342 ***	0.353 ***	0.442 ***	0.457 ***	0.479 ***

Note: * *p* < 0.05, ** *p* < 0.01, *** *p* < 0.001. Standardized coefficients were reported.

**Table 5:** Bootstrapping test for mediation effects (N = 432).

Paths	Estimates	S.E.	*p*-Values	95% CI
CES → PEE → TI	−0.050	0.020	0.012	[−0.090, −0.011]
CES → POG → TI	0.155	0.031	0.000	[0.095, 0.215]

**Table 6:** Bootstrapping test for moderated mediation effects (N = 432).

Paths	Organizational Identification	Estimates	S.E.	95% CI
CES → PEE → TI	Low (mean—1 SD)	−0.209	0.056	[−0.319, −0.117]
	High (mean + 1 SD)	−0.146	0.036	[−0.217, −0.086]
	Differences between low and high	0.063	0.021	[0.021, 0.098]
CES → POG → TI	Low (mean—1 SD)	0.059	0.043	[−0.024, 0.129]
	High (mean + 1 SD)	0.096	0.030	[0.007, 0.136]
	Differences between low and high	0.037	0.018	[0.001, 0.067]

## Data Availability

The data used in this research are available on request from the corresponding author. The data are not publicly available due to privacy issues.
